# *Fasciola* Metacercaria Contamination in Wetland Pastures and Its Association With Forage Characteristics Across Agro‐Ecological Zones: Case of Gamo Zone, Southern Ethiopia

**DOI:** 10.1002/vms3.71003

**Published:** 2026-05-28

**Authors:** Misganu Milkyas, Yisehak Kechero, Tamirat Kaba, Ephrem Tora, Gesila Endashaw, Tsegaye Yehoannes, Nigatu Eligo

**Affiliations:** ^1^ Department of Animal and Range Sciences Wolaita Sodo University Wolaita Sodo Ethiopia; ^2^ Department of Animal Sciences College of Agricultural Sciences Arba Minch University Arba Minch Ethiopia; ^3^ Department of Public Health College of Medicine and Health Sciences Arba Minch University Arba Minch Ethiopia

**Keywords:** agro‐ecology, ethiopia, fasciolosis, forage palatability, metacercariae, wetland pasture

## Abstract

Fasciolosis remains a major constraint to ruminant production in Ethiopia, particularly in wetland‐based grazing systems where infective *Fasciola* metacercariae encyst on pasture vegetation. This study investigated metacercarial contamination of wetland herbage and its association with forage characteristics across lowland, midland and highland agro‐ecologies of the Gamo Zone, southern Ethiopia. A cross‐sectional study was conducted from November 2021 to October 2022, during which 432 herbage samples were collected for parasitological and botanical analysis, and 171 livestock owners were interviewed regarding grazing practices. Metacercariae were recovered using a modified mechanical elution technique and expressed as cysts per kilogram of fresh herbage. Metacercarial burden varied significantly among herbaceous species and agro‐ecological zones (*p* < 0.05), with the highest contamination recorded in lowland (>750 cysts/kg) and highland (>600 cysts/kg) wetlands. Highly palatable and nutritionally superior species, including *Trifolium burchellianum*, *Cynodon nlemfuensis* and *Scirpus maritimus*, harboured the greatest metacercarial loads, demonstrating a positive association between forage quality and contamination risk. Most respondents (97.1%) relied on natural wetlands for grazing, and grazing management practices differed significantly among agro‐ecologies (*p* < 0.05). The findings indicate that wetland pastures in the Gamo Zone are important transmission foci for fasciolosis and that the concurrence of high nutritional value and elevated parasite contamination presents a critical challenge for livestock production. Integrated control strategies combining targeted grazing management and strategic anthelmintic treatment are recommended to reduce infection risk while maintaining feed availability.

## Graphical abstract

In Ethiopia's Gamo Zone, we found that highly palatable, protein‐rich forage species in wetlands carry the highest burden of *Fasciola* metacercariae. This creates a direct conflict between livestock nutrition and health, demanding integrated management strategies to mitigate disease risk.

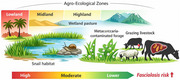

## 1 Introduction

Livestock production remains a cornerstone of Ethiopia's agrarian economy, contributing substantially to household income, food security and national GDP (Shapiro et al. 2017; CSA 2022; FAO 2023). However, its productivity is severely constrained by a combination of parasitic diseases and nutritional deficiencies. Among parasitic infections, fasciolosis, caused by liver flukes of the genus *Fasciola*, poses one of the most significant threats to livestock health and productivity. The disease leads to considerable economic losses through reduced growth and milk yield, decreased fertility and condemnation of infected livers at slaughter (Sargison and Scott 2011; FAO 2023).

The epidemiology of fasciolosis is closely linked to environmental conditions that favour the survival of its intermediate host, freshwater snails. Wetland pastures, which serve as essential feed resources during dry seasons, simultaneously provide ideal habitats for these snails (Biowott et al. 2025; Neira et al. 2025). The infective stage of the parasite, the metacercaria, encysts on aquatic and semi‐aquatic plants. When grazing animals ingest contaminated herbage, they become infected (Cuthill et al. 2020). This creates a paradox in livestock feeding systems: The very forages that sustain animals during critical feed shortages are also a major source of infection.

The Gamo Zone in southern Ethiopia exemplifies this paradox. The area is characterized by diverse agro‐ecological zones, lowland, midland and highland, with extensive seasonal wetlands and water‐logged pastures that are heavily grazed by livestock throughout the year (Temesgen et al. 2021). Despite the widespread occurrence of fasciolosis in the region, limited research has examined the specific association between wetland vegetation, nutritional characteristics of forages and the degree of contamination by *Fasciola* metacercaria. Understanding this relationship is crucial for balancing the nutritional benefits of wetland grazing with the need to mitigate parasitic infection risks. Moreover, the distribution of metacercaria on pasture is influenced by multiple interacting factors, including microclimate, snail density and plant species composition. Certain forage species may be more susceptible to contamination because of their leaf morphology, surface moisture retention or palatability to both snails and livestock (Kunta 2025). However, no systematic data exist for the Gamo Zone to identify which herbaceous species contribute most to the transmission of fasciolosis across different agro‐ecological gradients.

Therefore, this study was undertaken to investigate the extent of *Fasciola* metacercarial contamination in herbaceous species collected from natural wetland pastures across lowland, midland and highland agro‐ecologies of the Gamo Zone, Ethiopia. It also aimed to relate the burden of contamination to forage characteristics and palatability. The findings are expected to provide evidence‐based insights to inform integrated parasite control strategies and sustainable feed management practices in wetland‐dependent livestock systems.

## 2 Materials and Methods

### 2.1 The Study Area

**FIGURE 1:**
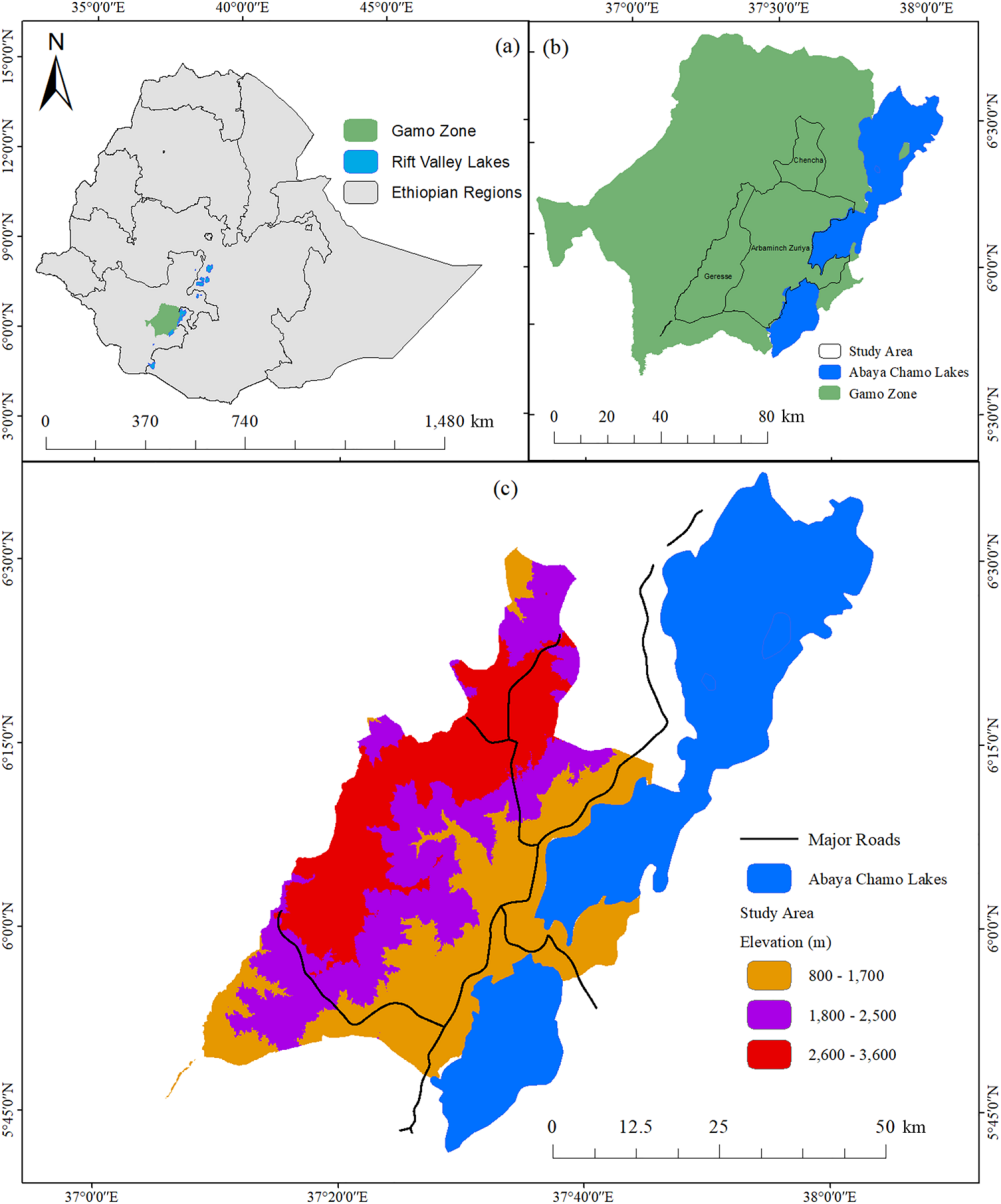
Location and agro‐ecological features of the study area in Gamo Zone. (a) Map of Ethiopia showing the location of Gamo Zone and the Rift Valley lakes region. (b) Administrative map of Gamo Zone indicating the study districts and the surrounding Abaya‐Chamo Lakes. (c) Elevation distribution and major road networks within the study area, showing the different agro‐ecological zones across the sampled wetland pastures.

The study was conducted in the wetland pastures of Gamo Zone, Southern Ethiopia. Sampling was stratified across three agro‐ecological zones—lowland (Arba Minch Zuria district), midland (parts of Geresse district) and highland (Chencha district)—to capture environmental variation influencing parasite transmission (Figure 1). The altitudinal range extends from approximately 1200 m in the lowlands to 3000 m above sea level in the highlands. The average temperature in the highlands is 16°C, whereas the average temperature in the lowlands is about 29°C. The average amount of rain that falls each year is between 800 and 1000 mm. The area experiences a bimodal rainfall pattern, with wet seasons from March to May and September to November.

Perennial water bodies, hydromorphic soils and dense vegetation support extensive wetland pastures, which serve as crucial dry‐season feed resources for livestock and provide favourable conditions for parasite survival and transmission.

### 2.2 Study Design and Sampling Strategy

A cross‐sectional study was conducted from November 2021 to October 2022 to assess the burden of *Fasciola* metacercariae in wetland pastures across lowland, midland and highland agro‐ecological zones. This design provides a snapshot of parasitic distribution and allows ecological associations to be examined under natural field conditions (Thrusfield 2018).

Sampling was stratified by agro‐ecology to capture environmental variability affecting trematode transmission, including differences in temperature, moisture and snail habitats (Yilma and Malone 1998; Mas Coma et al. 2005). Within each zone, three representative wetland grazing sites were randomly selected based on grazing intensity, persistent moisture, vegetation cover and proximity to water bodies—factors known to influence *Fasciola* transmission.

At each site, a botanical survey identified dominant and commonly grazed herbaceous species. For each species, three replicate herbage samples (5–10 g of leaves and tender stems clipped 2 cm above ground) were collected per site following standard parasitological procedures (Soulsby 1982; Hammond and Sewell 1990). A total of 432 samples were collected, evenly distributed among agro‐ecological zones. All samples were stored in labelled polyethylene bags, kept at 4–8°C during transport, and processed promptly in the laboratory to preserve metacercarial integrity (Mas Coma et al. 2005).

### 2.3 Recovery and Identification of *Fasciola* Metacercaria

Metacercariae were recovered using a modified mechanical elution method described by Cuthill et al. (2020). Approximately 5 g of herbage from each sample was soaked in 5 mL of dilute vinegar solution (prepared by adding 100 mL of vinegar to 1 L of distilled water) for 15 min to facilitate cyst detachment. The mixture was shaken vigorously and centrifuged manually using a salad spinner for about 1 min. The resulting fluid was strained through a 100 µm laboratory sieve and allowed to sediment for 10 min in a test tube. The supernatant was decanted, and the sediment volume was adjusted to 1 mL. The entire sediment was examined under a stereo microscope at low magnification. *Fasciola* metacercariae were identified morphologically by their spherical cyst shape, thick transparent wall and size, following the descriptions of Phalee et al. (2015). The total count of metacercariae was recorded and expressed as the number of cysts per kilogram of fresh herbage.

### 2.4 Botanical Identification and Palatability Assessment

Simultaneous with herbage sampling, botanical identification and palatability assessments were conducted at each site. Plant specimens were identified with the assistance of taxonomists from the Arba Minch University. The palatability of each herbaceous species was classified into four categories, highly palatable, palatable, less palatable and unpalatable, based on key informant interviews with experienced livestock owners and local experts. The classifications were validated against previous studies and direct field observations of grazing behaviour.

### 2.5 Farmer Survey on Pasture Utilization and Management

To complement the field data, a structured questionnaire was administered to livestock owners across the three agro‐ecological zones. The survey gathered information on pasture availability, grazing management systems, traditional conservation practices and local grass species used for livestock feeding. A total of 171 respondents were interviewed (58 from lowland, 51 from midland and 62 from highland areas), selected through simple random sampling to represent active livestock keepers with knowledge of wetland grazing practices.

### 2.6 Data Management and Statistical Analysis

All quantitative data were entered into Microsoft Excel, checked for completeness and consistency, and subsequently analysed using R software (version 4.1.0) (R Core Team 2021). Metacercarial burden, expressed as the number of metacercariae per kilogram of herbage, constituted continuous response data. Prior to inferential analysis, the distributional properties of the data were evaluated. Normality of residuals was assessed using the Shapiro–Wilk test and visual inspection of Q–Q plots, whereas homogeneity of variances was examined using Levene's test. Because metacercarial burden data exhibited right‐skewness typical of parasitological count data, a logarithmic transformation was applied to stabilize variances and approximate normality, in line with recommended procedures for ecological and parasitological datasets (Zuur et al. 2009; Thrusfield 2018).

After confirming that model assumptions were adequately satisfied post‐transformation, parametric statistical tests were performed. Differences in mean metacercarial burden among herbaceous species and agro‐ecological zones were analysed using one‐way analysis of variance (ANOVA), followed by Tukey's Honestly Significant Difference (HSD) test for pairwise comparisons when significant effects were detected, with statistical significance declared at *p* < 0.05. Correlation analysis was conducted to evaluate the association between crude protein (CP%) and metacercarial burden after verifying linearity and normality assumptions. Descriptive statistics summarized survey variables, and chi‐square (*χ* ^2^) tests assessed differences among agro‐ecological zones for categorical variables.

## 3 Results

### 3.1 Traditional Grazing Management and Local Grass Distribution

Natural grazing wetlands were reported by the majority of respondents across agro‐ecological zones, including the lowland (94.8%), midland (96.1%) and highland (100%) areas (Table 1). No statistically significant difference was observed among zones (*χ* ^2^ = 3.079, *p* = 0.059), indicating that natural pastures constitute the principal livestock feed resource throughout the Gamo Zone.

**TABLE 1:** Traditional grazing management practices and distribution of local grass species across agro‐ecological zones.

Variables	Categories/Species	Lowland (*n* = 58)	Midland (*n* = 51)	Highland (*n* = 62)	Total (*N* = 171)	*χ* ^2^	*p* value
Natural grazing wetland (%)	Yes	94.8	96.1	100.0	97.1	3.079	0.059
	No	5.2	3.9	0.0	2.9		
Traditional management practices (%)	Yes	60.3	88.2	96.8	81.9	28.770	<0.001
	No	39.7	11.8	3.2	18.1		
Type of grazing management (%)	Enclosing of grazing land	5.2	21.6	16.1	14.0	21.360	0.002
	Rotational grazing	50.0	23.5	16.1	29.8		
	Communal grazing	31.0	43.1	50.0	41.5		
	Free grazing land	13.8	11.8	17.7	14.6		
Management of grazing land (%)	Fertilizer application	0.0	0.0	0.8	0.2	25.400	0.001
	Manure application	89.7	74.5	89.5	84.5		
	Weeding	10.3	24.5	1.6	12.1		
	No management	0.0	1.5	8.1	3.2		
Local grass species (%)	*Cynodon nlemfuensis* (Sura)	26.3	37.3	44.3	36.1	19.350	0.036
	*Acroceras macrum* (Aba mataa)	8.8	23.5	16.4	16.0		
	*Sporobolus fertilis* (Xinka)	31.6	27.4	18.0	25.6		
	*Bolboschoenus maritimus* (Chafee)	17.5	2.0	13.1	11.2		
	*Scirpus maritimus* (Michaa)	7.8	2.0	1.6	3.8		
	*Loudetia kagerensis* (Qoli mataa)	7.0	5.9	6.6	6.5		
	*Centella asiatica* (Qophando)	0.0	0.0	1.5	0.5		
	*Typha angustifolia* (Dolikuwa)	0.0	1.0	0.0	0.3		
	*Commelina benghalensis* (Dalisha)	0.5	0.0	0.0	0.1		

*Note*: Values are percentages of respondents within each agro‐ecological zone; *χ* ^2^ = chi‐square test statistic. Statistical significance declared at ***p* ≤ 0.05**.

Traditional grazing management practices were employed by 81.9% of respondents overall, with significant variation among agro‐ecological zones (*χ* ^2^ = 28.77, *p* < 0.001). Adoption was lowest in the lowlands (60.3%), followed by the midlands (88.2%), and highest in the highlands (96.8%). Grazing systems differed significantly across zones (*χ* ^2^ = 21.36, *p* = 0.002). Communal grazing was the predominant system (41.5%), particularly in highland (50.0%) and midland (43.1%) areas, whereas rotational grazing was more frequently reported in the lowlands (50.0%). Free grazing and enclosed grazing systems accounted for 14.6% and 14.0% of responses, respectively.

Management interventions applied to grazing lands varied significantly among zones (*χ* ^2^ = 25.40, *p* = 0.001). The majority of respondents (84.5%) indicated the use of animal manure to enhance pasture fertility, with higher frequencies in lowland (89.7%) and highland (89.5%) areas. Weeding was primarily practiced in the midlands (24.5%), whereas the application of chemical fertilizers was rarely reported across all zones. A small proportion of respondents (3.2%), predominantly from highland areas, indicated the absence of any grazing land management practices. The occurrence of local grass species differed significantly among agro‐ecological zones (*χ* ^2^ = 19.35, *p* = 0.036). The most frequently reported species were *Cynodon nlemfuensis* (locally known as Sura; 36.1%), *Sporobolus fertilis* (Xinka; 25.6%) and *Acroceras macrum* (Aba mata; 16.0%). *C. nlemfuensis* was more commonly observed in highland areas (44.3%), whereas *Acroceras macrum* was more prevalent in midland zones (23.5%). Lowland wetlands were predominantly characterized by *Bolboschoenus maritimus* (Chafee) and *Scirpus maritimus* (Michaa), reflecting species adapted to seasonally waterlogged environments.

### 3.2 Burden of Metacercaria in Herbaceous Species From the Lowland Agro‐Ecology

The metacercaria burden recorded on selected herbaceous species in the lowland agro‐ecology of Gamo zone is presented in Table 2. A total of seven herbaceous species representing different palatability classes were examined. Among the highly palatable species, *Loudetia kagerensis* recorded the highest mean metacercaria burden (782.5 no./kg herbage), with values ranging from 410 to 1340 no./kg. This was followed by *A. macrum* (mean = 548.2 no./kg; range: 220–980) and *B. maritimus* (mean = 472.6 no./kg; range: 180–820). Similarly, *C. nlemfuensis* showed a relatively high mean burden (401.3 no. /kg), although with a wide range (115–890).

**TABLE 2:** Metacercaria burden on selected herbaceous species in lowland agro‐ecology.

Herbaceous species	Palatability class	*n* (observations)	Mean burden (no./kg herbage)	Minimum	Maximum
*Loudetia kagerensis*	Highly palatable	12	782.5	410	1340
*Acroceras macrum*	Highly palatable	10	548.2	220	980
*Bolboschoenus maritimus*	Highly palatable	9	472.6	180	820
*Cynodon nlemfuensis*	Highly palatable	12	401.3	115	890
*Sporobolus fertilis*	Palatable	11	356.4	140	640
*Leptochloa chinensis*	Highly palatable	8	309.7	95	520
*Hyparrhenia tamba*	Less palatable	10	228.9	60	410

*Note*: Overall ANOVA *p* value: 0.074 (not significant).

The palatable species *S. fertilis* had a moderate mean burden of 356.4 no./kg (range: 140–640). In contrast, *Leptochloa chinensis*, although categorized as highly palatable, showed a comparatively lower mean burden (309.7 no./kg; range: 95–520). The least palatable species, *Hyparrhenia tamba*, recorded the lowest mean burden (228.9 no./kg), with values ranging from 60 to 410.

### 3.3 Burden of Metacercaria in Herbaceous Species in Midland Agro‐Ecology

The metacercaria burden on selected herbaceous species in the midland agro‐ecology is presented in Table 3. In contrast to the lowland findings, statistically significant differences were observed among plant species (one‐way ANOVA, *p* = 0.026). Among the highly palatable species, *Trifolium burchellianum* recorded the highest mean metacercaria burden (612.4 no./kg herbage; range: 340–980), followed by *L. chinensis* (528.7 no./kg; 260–910). The less palatable *Bolboschoenus glaucus* showed a relatively high mean burden (463.2 no./kg; 190–770). Among the palatable species, *Cymbopogon giganteus and S. fertilis* exhibited moderate contamination levels, with mean burdens of 409.5 no./kg (170–690) and 352.8 no./kg (140–560), respectively. The lowest mean burden was recorded in the unpalatable *Typha angustifolia* (72.3 no./kg; 10–140), followed by *S. maritimus* (298.6 no./kg; 110–480). Overall, highly palatable species demonstrated significantly greater metacercaria burdens compared with less palatable and unpalatable species.

**TABLE 3:** Metacercaria burden on selected herbaceous species in midland agro‐ecology.

Herbaceous species	Palatability class	*n* (observations)	Mean burden (no./kg herbage)	Minimum	Maximum
*Trifolium burchellianum*	Highly palatable	12	612.4^a^	340	980
*Leptochloa chinensis*	Highly palatable	10	528.7^b^	260	910
*Bolboschoenus glaucus*	Less palatable	9	463.2^c^	190	770
*Cymbopogon giganteus*	Palatable	11	409.5^d^	170	690
*Sporobolus fertilis*	Palatable	10	352.8^e^	140	560
*Scirpus maritimus*	Palatable	8	298.6^f^	110	480
*Typha angustifolia*	Unpalatable	9	72.3^g^	10	140

*Note*: Overall ANOVA *p* value: 0.026 (significant).

Values with different superscript letters (a‐g) within the column differ significantly at *p* < 0.05 according to Tukey's HSD post hoc test.

### 3.4 Burden of Metacercaria in Herbaceous Species in Highland Agro‐Ecology

The metacercaria burden on selected herbaceous species in the highland agro‐ecology is presented in Table 4. Although numerical variation in mean burden was observed among plant species, the overall difference was not statistically significant (one‐way ANOVA, *p* = 0.074). Among the examined species, *S. maritimus* (palatable) recorded the highest mean metacercaria burden (628.5 no./kg herbage; range: 320–990), followed by the less palatable *B. glaucus* (574.3 no./kg; 260–910). *Hyparrhenia rufa* (palatable) showed a mean burden of 503.6 no./kg (210–820). The highly palatable *C. nlemfuensis* recorded a mean burden of 452.8 no./kg (140–880), whereas the unpalatable *Centella asiatica* showed a relatively high mean contamination (398.4 no./kg; 120–690). The highly palatable *L. kagerensis* had a mean burden of 346.7 no./kg (95–620). The lowest burden was observed in the less palatable *Cyperus sanguinolentus* (182.9 no./kg; 40–360). Overall, although palatable species tended to exhibit moderate to high contamination levels, metacercaria burden did not differ significantly among species in the highland agro‐ecology.

**TABLE 4:** Metacercaria burden on selected herbaceous species in highland agro‐ecology.

Herbaceous species	Palatability class	*n* (observations)	Mean burden (no./kg herbage)	Minimum	Maximum
*Scirpus maritimus*	Palatable	11	628.5	320	990
*Bolboschoenus glaucus*	Less palatable	9	574.3	260	910
*Hyparrhenia rufa*	Palatable	10	503.6	210	820
*Cynodon nlemfuensis*	Highly palatable	12	452.8	140	880
*Centella asiatica*	Unpalatable	8	398.4	120	690
*Loudetia kagerensis*	Highly palatable	10	346.7	95	620
*Cyperus sanguinolentus*	Less palatable	9	182.9	40	360

*Note*: Overall ANOVA *p* value: 0.074 (not significant).

### 3.5 Metacercaria Burden by Plant Palatability: Higher Contamination in Livestock‐Preferred Species

The relationship between plant palatability and mean metacercaria burden is summarized in Table 5. A clear gradient in metacercaria burden was observed across palatability classes. Highly palatable species, represented by *C. nlemfuensis* and *T. burchellianum*, consistently exhibited high to very high mean metacercaria burdens across agro‐ecological zones. Palatable species such as *S. fertilis* and *S. maritimus* showed moderate to high burdens. In contrast, less palatable or unpalatable species, including *H. tamba* and *T. angustifolia*, were associated with low metacercaria burdens. Overall, metacercaria burden showed a positive association with plant palatability, with higher contamination levels recorded on species preferred by grazing livestock.

**TABLE 5:** Relationship between plant palatability and mean metacercaria burden.

Palatability class	Example species	General metacercaria burden trend	Inferred reason
Highly palatable	*Cynodon nlemfuensis* and *Trifolium burchellianum*	High to very high	Preferred by both snails and livestock; tender leaves ideal for encystment
Palatable	*Sporobolus fertilis* and *Scirpus maritimus*	Moderate to high	Attractive to grazing animals, providing a route for infection
Less palatable/Unpalatable	*Hyparrhenia tamba* and *Typha angustifolia*	Low	Physical (hard leaves) or chemical traits deter snails and/or metacercarial attachment

### 3.6 Trade‐Offs Between CP Content and Metacercariae Burdens in Key Forage Species Across Agro‐Ecologies

The nutritional quality and contamination risk of four forage species collected from distinct agro‐ecological zones are summarized in Table 6. *T. burchellianum* sampled from the midland exhibited a CP concentration of 12.76%, categorizing it as a high‐quality forage. However, this species concurrently harboured a mean metacercaria burden exceeding 578 no./kg, indicating a very high fasciolosis risk. Similarly, *S. maritimus* from the highland zone demonstrated high nutritive value with 11.86% CP, yet presented an even greater parasite load (>600 no./kg). *C. nlemfuensis*, also from the highland region, had the highest measured nutritional quality among the assessed species (13.29% CP, very high), but was associated with a high mean metacercaria burden of approximately 450 no./kg. In contrast, *T. angustifolia* from the midland showed a low mean metacercaria burden (<88 no./kg), reflective of low contamination risk; its nutritional quality was not determined. These patterns demonstrate that the forage species with the greatest CP content were also those most heavily contaminated with infective trematode stages.

**TABLE 6:** Comparison of crude protein content and fasciolosis risk of key forage species across agro‐ecologies.

Species	Agro‐ecology	Crude protein (CP, %)^1^	Mean metacercaria burden (no./kg)^2^	Implication
*Trifolium burchellianum*	Midland	12.76 (high)	>578 (very high)	High‐quality forage with very high fasciolosis risk
*Scirpus maritimus*	Highland	11.86 (high)	>600 (very high)	Nutritious sedge posing major health threat
*Cynodon nlemfuensis*	Highland	13.29 (very high)	∼450 (high)	Most nutritious grass but heavily contaminated
*Typha angustifolia*	Midland	Not analysed	<88 (low)	Low feed value but minimal fasciolosis risk

*Note*: CP = Crude protein, an indicator of forage nutritive quality. Values in parentheses reflect qualitative classification: High = >11%, very high = >13%; mean metacercaria burden refers to the number of infective *Fasciola* metacercariae per kg of forage. Classification: low = <100, high = 300–500 and very high = >500.

1 Crude protein (CP) values obtained from forage nutritional analysis.

2 Mean metacercaria burden expressed as the number of infective Fasciola metacercariae per kilogram of fresh forage.

### 3.7 Cross‐Agro‐Ecological Zone Comparison of Forage Protein Content Versus Fasciolosis Risk

The nutritional quality and metacercarial contamination of 21 forage species across lowland, midland and highland agro‐ecological zones are summarized in Table 7. Lowland species such as *L. kagerensis* and *A. macrum* exhibited moderate protein content (10.5%–11.2% CP) but carried high parasite burdens (500–750 no./kg). *C. nlemfuensis* maintained the highest protein content among lowland species (13.2% CP) while still presenting a significant metacercarial load (400 no./kg). Midland forages, including *T. burchellianum*, displayed very high protein levels (14.8% CP) but concurrently had very high contamination (578 no./kg), whereas *T. angustifolia* showed minimal parasite risk (<88 no./kg) but low nutritional value (5.2% CP).

**TABLE 7:** Protein content and metacercarial burden of key forage species across agro‐ecological zones.

Species	Agro‐ecology	Mean metacercaria burden (no./kg)^1^	CP (%)	Implication
*Loudetia kagerensis*	Lowland	750	10.5	High parasite risk with moderate protein
*Acroceras macrum*	Lowland	500	11.2	Moderate nutritional value; high contamination
*Bolboschoenus maritimus*	Lowland	450	9.4	Low‐moderate protein; high parasite risk
*Cynodon nlemfuensis*	Lowland	400	13.2	High protein; significant parasite risk
*Sporobolus fertilis*	Lowland	350	8.5	Low protein; moderate parasite load
*Leptochloa chinensis*	Lowland	300	10.8	Moderate protein; moderate parasite load
*Hyparrhenia tamba*	Lowland	250	6.8	Low protein; moderate parasite risk
*Trifolium burchellianum*	Midland	578	14.8	Very high protein; very high parasite risk
*Leptochloa chinensis*	Midland	500	10.8	Moderate protein; high parasite risk
*Bolboschoenus glaucus*	Midland	450	7.5	Low protein; high contamination
*Cymbopogon giganteus*	Midland	400	7.8	Low protein; moderate parasite risk
*Sporobolus fertilis*	Midland	350	8.5	Low protein; moderate parasite risk
*Scirpus maritimus*	Midland	300	10.6	Moderate protein; moderate contamination
*Typha angustifolia*	Midland	88	5.2	Low protein; minimal parasite risk
*Scirpus maritimus*	Highland	600	10.6	Moderate protein; very high parasite risk
*Bolboschoenus glaucus*	Highland	550	7.5	Low protein; very high contamination
*Hyparrhenia rufa*	Highland	500	8.2	Low‐moderate protein; high parasite risk
*Cynodon nlemfuensis*	Highland	450	13.2	High protein; high contamination
*Centella asiatica*	Highland	400	6.5	Low protein; moderate parasite risk
*Loudetia kagerensis*	Highland	350	10.5	Moderate protein; moderate parasite load
*Cyperus sanguinolentus*	Highland	200	7.1	Low protein; low parasite risk

*Note*: Mean metacercaria burden = number of infective *Fasciola* metacercariae per kg of forage. Classification: low (<100), moderate (100–400), high (401–600) and very high (>600); hypothetical CP (%) represents estimated crude protein content as an indicator of nutritional value.

1 Mean metacercaria burden expressed as the number of infective Fasciola metacercariae per kilogram of fresh forage.

Highland species demonstrated a similar pattern, with highly nutritious species like *C. nlemfuensis* (13.2% CP) exhibiting elevated metacercarial burdens (∼450 no./kg). Overall, species with the greatest nutritive value consistently carried higher metacercarial contamination, highlighting the trade‐off between feed quality and fasciolosis risk.

### 3.8 Spearman's Rank Correlation Between CP Content and Metacercarial Burden in Forage Species

Spearman's rank correlation analysis was performed to assess the relationship between CP content and metacercarial burden across 21 forage species (Table 8). The analysis yielded a moderate positive correlation (*ρ* = 0.495, *n* = 21, *p* = 0.022), indicating that forage species with higher CP content tended to have higher metacercarial burdens.

**TABLE 8:** Spearman rank calculation for the relationship between crude protein (CP) content and metacercarial burden of forage species (*n* = 21).

Obs.	CP (%)	CP (Rank)	Metacercarial burden	Rank (burden)	*d*	*d* ^2^
1	10.5	13.5	750	21.0	−7.5	56.25
2	11.2	15.0	500	16.0	−1.0	1.00
3	9.4	9.0	450	13.0	−4.0	16.00
4	13.2	19.5	400	10.0	9.5	90.25
5	8.5	7.5	350	7.5	0.0	0.00
6	10.8	14.5	300	5.5	9.0	81.00
7	6.8	3.0	250	3.0	0.0	0.00
8	14.8	21.0	578	18.0	3.0	9.00
9	10.8	14.5	500	16.0	−1.5	2.25
10	7.5	5.5	450	13.0	−7.5	56.25
11	7.8	6.0	400	10.0	−4.0	16.00
12	8.5	7.5	350	7.5	0.0	0.00
13	10.6	12.0	300	5.5	6.5	42.25
14	5.2	1.0	88	1.0	0.0	0.00
15	10.6	12.0	600	19.0	−7.0	49.00
16	7.5	5.5	550	17.0	−11.5	132.25
17	8.2	7.0	500	16.0	−9.0	81.00
18	13.2	19.5	450	13.0	6.5	42.25
19	6.5	2.0	400	10.0	−8.0	64.00
20	10.5	13.5	350	7.5	6.0	36.00
21	7.1	4.0	200	2.0	2.0	4.00
**Σ**	—	—	—	—	—	**778.00**

*Note*: CP (%) = crude protein percentage in forage samples; CP = rank of crude protein values used for Spearman rank correlation analysis; metacercarial burden = number of *Fasciola* metacercariae detected on forage samples; ranks were assigned in ascending order (lowest value = 1). Tied values were assigned mean ranks; *d* = difference between ranks (rank CP − rank burden); Σ*d* ^2^ = 778; Spearman's rank correlation coefficient was calculated as *ρ* = 0.495 (*n* = 21, *p* = 0.022).

The rank differences (*d*) between CP and metacercarial burden varied widely among species, ranging from −11.5 to 9.5, and the sum of squared rank differences (Σ*d* ^2^) was 778. Species with the highest CP values, such as *T. burchellianum* (21.0 rank) and *C. nlemfuensis* (19.5 rank), corresponded with relatively high ranks for metacercarial burden, whereas low‐protein species, such as *T. angustifolia* (1.0 rank) and *C. asiatica* (2.0 rank), exhibited low parasite loads. The *p* value (0.022) indicates that this correlation is statistically significant at the 5% level, supporting the inference that nutritional value and fasciolosis risk are positively associated in these forage species.

## 4 Discussion

### 4.1 Traditional Grazing Management and Local Grass Distribution

Natural grazing wetlands dominate across lowland (94.8%), midland (96.1%) and highland (100%) agro‐ecological zones, confirming that natural pastures are the primary livestock feed resource in the Gamo Zone. This widespread reliance aligns with previous studies in East African mixed crop–livestock systems, where communal rangelands underpin smallholder livestock production (Tefera et al. 2018; Desta and Coppock 2020). The absence of significant differences in grazing wetland availability among zones (*χ* ^2^ = 3.079, *p* = 0.059) suggests that environmental gradients do not restrict pasture access, although seasonal variability may influence forage quantity and quality (Bekele and Fetene 2021).

Traditional grazing management practices were reported by 81.9% of respondents, with significant zonal variation (*χ* ^2^ = 28.77, *p* < 0.001). Adoption was lower in lowlands (60.3%) due to extensive roaming and opportunistic grazing, whereas midlands and highlands showed higher adoption, likely driven by land scarcity, higher livestock density and cultural norms reinforcing customary grazing rules (Seyoum et al. 2021; Abebe et al. 2019; Mekonnen and Beshah 2022). Grazing systems also varied (*χ* ^2^ = 21.36, *p* = 0.002), with communal grazing dominating in highland (50.0%) and midland (43.1%) zones, whereas rotational grazing was more frequent in lowlands (50.0%). These practices influence pasture sustainability, as communal grazing allows shared access but may cause overgrazing, whereas rotational grazing can improve regeneration when effectively implemented (Shapiro et al. 2020; Yohannes et al. 2022; Tefera et al. 2019).

Management interventions and grass species composition differed significantly among zones (*χ* ^2^ = 25.40, *p* = 0.001; *χ* ^2^ = 19.35, *p* = 0.036). Manure application was the most common practice (84.5%), supporting soil fertility, whereas weeding (24.5%) and chemical fertilizers were less frequent (Teshome and Smit 2022; Erkossa et al. 2020; Yilma and Dessalegn 2021). Grass species distribution reflected ecological adaptation: *C. nlemfuensis* and *S. fertilis* were prevalent in highlands, *A. macrum* in midlands and *B. maritimus* and *S. maritimus* dominated lowlands (Unger et al. 2019; Legesse and Alemayehu 2023; Mengistu and Abebe 2020). Integrating traditional management with science‐based interventions, such as rotational grazing, controlled rest periods and seedbanks for ecologically adapted species, may enhance pasture productivity and livestock nutrition across the Gamo Zone (Tefera et al. 2022).

### 4.2 Variation in Metacercariae Burdens Among Herbaceous Species in Lowland Agro‐Ecologies

Variation in metacercarial burdens was evident among herbaceous species in the lowland agro‐ecology, with numerically higher contamination observed in highly palatable grasses. Although interspecies differences did not reach statistical significance, the trends remain biologically meaningful for both parasitology and livestock management. Preferential grazing on palatable forage may increase the likelihood of ingesting encysted metacercariae, thereby elevating the risk of trematode infections such as fasciolosis and paramphistomosis (Soulsby 1982; Urquhart et al. 1996). The comparatively higher burden detected in *L. kagerensis* could be attributed to species‐specific morphological traits and favourable microenvironments that support cercarial attachment and survival.

Trematode transmission is strongly influenced by ecological and climatic factors, including the availability of suitable snail intermediate hosts and moist habitats conducive to larval development (Mas‐Coma et al. 2005; Taylor et al. 2016). Lowland areas typically provide optimal conditions for snail vectors due to elevated temperatures, seasonal water accumulation and sustained humidity. Although differences were not statistically significant, even moderate variation in pasture contamination can contribute to subclinical infections, impaired productivity and economic losses.

These findings highlight the importance of integrated trematode control strategies in lowland agro‐ecologies. Effective measures may include pasture management, reduction of snail habitats, strategic grazing and appropriately timed anthelmintic interventions (Ollerenshaw and Rowlands 1959; Fox et al. 2011; Charlier et al. 2014). Monitoring species‐specific contamination patterns can provide actionable insights for veterinary practitioners and livestock managers.

### 4.3 Herbaceous Species Determine Metacercaria Burden in Midland Agro‐Ecologies

In the midland agro‐ecology, a statistically significant association was observed between herbaceous species and metacercarial burden (*p* = 0.026). Highly palatable species such as *T. burchellianum* and *L. chinensis* exhibited the highest contamination, whereas the less palatable *T. angustifolia* demonstrated substantially lower burdens. These findings are epidemiologically important, as grazing preference for palatable species increases the likelihood of ingesting infective trematode stages (Soulsby 1982; Taylor et al. 2016; Charlier and Rinaldi 2022).

Morphological and ecological traits of plant species, including leaf toughness, growth habit and proximity to wet microhabitats, likely influence metacercarial deposition (Mas Coma et al. 2005; Fox et al. 2011). Wetland‐associated species such as *B. glaucus* and *S. maritimus* showed moderate to high contamination, reflecting the contribution of environmental factors alongside grazing behaviour. These observations underline the necessity of considering both plant characteristics and habitat conditions in epidemiological assessments.

From a livestock management perspective, preferential grazing on highly contaminated species can intensify cumulative infection pressure, leading to subclinical disease and productivity losses. Integrated control strategies combining pasture management, drainage of wet areas, snail habitat reduction and targeted anthelmintic treatment are recommended to mitigate infection risk and improve overall animal health (Megersa et al. 2024; Endalamew et al. 2025).

### 4.4 Herbaceous Species and Metacercaria Burdens in Highland Agro‐Ecology

In highland agro‐ecologies, no statistically significant interspecies differences were observed (*p* = 0.074), indicating a relatively uniform distribution of metacercariae across herbaceous vegetation. Nonetheless, numerical trends suggest that species such as *S. maritimus* and *B. glaucus* may disproportionately contribute to pasture contamination. These differences, although moderate, remain biologically relevant given that highland conditions, characterized by cooler temperatures, persistent moisture and frequent rainfall, favour the survival of trematode free‐living stages (Mas‐Coma et al. 2005; Taylor et al. 2016).

Wetland‐associated species occupy habitats conducive to snail intermediate hosts, facilitating cercarial emergence and encystment on surrounding vegetation (Ollerenshaw and Rowlands 1959; Fox et al. 2011). The elevated burden in less palatable species, such as *C. asiatica*, indicates that environmental exposure may be a stronger driver of contamination than grazing preference alone. This pattern supports the concept that microclimatic conditions can homogenize pasture contamination in highland systems.

Uniform contamination across plant species may result in continuous low‐to‐moderate infection pressure, contributing to subclinical infections and chronic production losses. These findings emphasize the continued relevance of integrated control strategies, including strategic anthelmintic administration, drainage management and snail habitat reduction, tailored to highland agro‐ecological conditions (Megersa et al. 2024; Endalamew et al. 2025).

### 4.5 How Forage Preferences Shape Trematode Infection Risk Across Agro‐Ecologies

The positive association between forage palatability and metacercarial burden demonstrates the combined influence of ecological and behavioural determinants on trematode transmission. Highly palatable species such as *C. nlemfuensis* and *T. burchellianum* consistently exhibited elevated contamination, suggesting that tender, nutrient‐rich foliage not only attracts grazing ruminants but also provides favourable microhabitats for cercarial encystment (Mereta et al. 2023; Megersa et al. 2024).

Moderate to high contamination in structurally distinct palatable species, such as *S. fertilis* and *S. maritimus*, further emphasizes the ecological link between vegetation characteristics and infection risk. Snail intermediate hosts commonly inhabit moist vegetation accessible to livestock, increasing the probability of cercarial deposition. Conversely, less palatable species, including *H. tamba* and *T. angustifolia*, exhibited lower metacercarial burdens due to structural and chemical traits that discourage grazing and reduce cercarial attachment (Hammoud et al. 2025).

These relationships underscore the importance of integrating vegetation characteristics into pasture and parasite management. Strategies such as rotational grazing, wetland management and targeted anthelmintic administration can effectively reduce trematode exposure while maintaining forage availability for livestock (Charlier et al. 2014; Mas‐Coma et al. 2005). Monitoring high‐risk forage species supports evidence‐based decision‐making in endemic agro‐ecologies.

### 4.6 High‐Quality Forage as a Double‐Edged Sword in Fasciolosis‐Endemic Grazing Systems

High‐quality forage species may simultaneously confer nutritional benefits and elevate fasciolosis risk. Species such as *C. nlemfuensis* and *T. burchellianum* were associated with higher burdens of infective *Fasciola* metacercariae, reflecting conditions favourable to both forage growth and trematode survival, including moist, marshy environments with abundant snail hosts (Mas‐Coma et al. 2005; Megersa et al. 2024).

Conversely, structurally robust or less palatable species, such as *T. angustifolia*, exhibited minimal contamination, reducing infection risk but potentially offering limited nutritional value. Plant morphology, vertical growth and habitat preference influence both cercarial attachment and grazing behaviour, creating a nutritional–parasitological trade‐off.

Veterinary and livestock management strategies must therefore balance productivity with infection risk. Measures include strategic grazing management to avoid high‐risk habitats, forage conservation or processing to reduce metacercarial viability and targeted anthelmintic interventions. Surveillance of forage contamination and mapping of snail vectors can inform evidence‐based grazing decisions and optimize livestock health in fasciolosis‐endemic systems (Soulsby 1982; Ali et al. 2025; Mathewos et al. 2023).

### 4.7 Trade‐Offs Between Forage Nutritive Value and Fasciolosis Risk Across Agro‐Ecological Zones

Across agro‐ecological zones, a consistent trade‐off exists between forage nutritive value and fasciolosis risk. Species with higher CP content, including *T. burchellianum* in the midlands and *C. nlemfuensis* across multiple zones, were associated with elevated metacercarial burdens, reflecting ecological conditions that support both forage productivity and trematode survival (Mas‐Coma et al. 2005; Hansen and Perry 1994).

Zone‐specific patterns demonstrate that moderate‐quality forage can still pose substantial infection risk, whereas species with minimal contamination often provide limited nutritional value. This illustrates the complex interplay between forage quality, environmental suitability and parasite transmission.

These findings emphasize the need for integrated management strategies that consider both nutritional and parasitological aspects. Approaches such as forage selection, habitat modification, rotational grazing and strategically timed anthelmintic interventions are recommended. Surveillance and mapping of high‐risk habitats further enhance evidence‐based decision‐making in fasciolosis‐endemic regions (Torgerson and Claxton 1999; Fennouh et al. 2025; Sargison et al. 2016).

### 4.8 Forage Protein Content as a Predictor of Metacercarial Burden

A moderate, statistically significant positive correlation between CP content and metacercarial burden (*ρ* = 0.495, *p* = 0.022) supports the existence of a nutritional–parasitological trade‐off. High‐protein forage promotes growth, reproduction and lactation in ruminants but is also associated with increased exposure to infective metacercariae due to favourable microhabitats for trematode survival and snail host proliferation (Mas‐Coma et al. 2005; Sargison 2012).

Low‐protein species, including *T. angustifolia* and *C. asiatica*, generally exhibited minimal contamination, reducing infection risk but limiting nutritional benefit. This indicates that forage quality and safety do not necessarily coincide in endemic systems.

These findings underscore the importance of integrated feeding and parasite control strategies. Forage selection should be accompanied by pasture monitoring, rotational grazing, conservation or processing methods and targeted anthelmintic treatment. Considering additional ecological factors such as vegetation structure and microhabitat characteristics further optimizes livestock health and productivity in fasciolosis‐endemic systems (Torgerson and Claxton 1999; Ali et al. 2025).

## 5 Conclusions

This study demonstrates that natural wetland pastures in Ethiopia's Gamo Zone are major transmission foci for *Fasciola* metacercariae, with contamination burdens varying significantly across agro‐ecologies and peaking in lowland and highland areas. A critical positive correlation was established between forage nutritive value and parasite load; highly palatable species with high CP content, including *T. burchellianum* and *C. nlemfuensis*, consistently harboured the greatest metacercarial burdens. This finding reveals a fundamental nutritional—parasitological trade‐off whereby the most valuable dry‐season forages present the highest infection risk—a dilemma exacerbated by over 97% farmer reliance on communal wetlands and ecological conditions favouring snail intermediate hosts. Sustainable livestock production therefore requires integrated, evidence‐based control strategies tailored to specific agro‐ecologies. We recommend combining targeted grazing management to restrict wetland access during peak transmission periods, strategic anthelmintic treatment synchronized with seasonal exposure patterns and community‐based habitat modification to reduce snail populations. These interventions should mitigate fasciolosis risk while preserving the essential role of wetland resources in mixed crop‐livestock systems.

## Author Contributions

**Misganu Milkyas**: conceptualization, methodology, investigation. **Yisehak Kechero**: conceptualization, methodology, validation, supervision, funding acquisition, visualization, writing – review and editing. **Tamirat Kaba**: conceptualization, methodology, data curation, investigation, writing – review and editing. **Ephrem Tora**: conceptualization, methodology, validation, formal analysis, supervision, visualization, writing – review and editing. **Gesila Endashaw**: conceptualization, methodology, investigation. **Tsegaye Yehoannes**: conceptualization, methodology, investigation. **Nigatu Eligo**: conceptualization, methodology, investigation. All authors have read and approved the final manuscript and agree to be accountable for all aspects of the work.

## Funding

This research was funded by Arba Minch University (Grant No. AMU/AnSc‐5/2024).

## Disclosure

The funding body had no role in the design of the study; in the collection, analysis or interpretation of data; or in writing the manuscript.

## Ethics Statement

Ethical approval for this study was obtained from the Research Ethics Review Committee of Arba Minch University, College of Agricultural Sciences (Approval No. 20/12/2024). The study involved livestock owners and was conducted in accordance with relevant national and international ethical guidelines.

## Consent

Participants were informed about the objectives of the study in their local language, and participation was voluntary. Verbal informed consent was obtained prior to data collection. No personal identifiers were recorded, and all data were handled confidentially and used solely for research purposes.

## Conflicts of Interest

The authors declare no conflicts of interest.

## Future Research Directions

Longitudinal studies are needed to quantify seasonal contamination patterns and transmission dynamics across multiple years. Molecular characterization of circulating *Fasciola* species and their snail intermediate hosts would clarify epidemiological relationships. Additionally, controlled trials evaluating forage preservation methods (haymaking and ensiling) for their efficacy in reducing metacercarial viability could provide practical intervention options. Finally, participatory action research assessing the socio‐economic feasibility and adoption potential of recommended management strategies would support evidence‐based policy development.

## Data Availability

The datasets generated and/or analysed during the current study are available from the corresponding author on reasonable request.
